# The radiosensitivity index predicts for overall survival in glioblastoma

**DOI:** 10.18632/oncotarget.5437

**Published:** 2015-10-03

**Authors:** Kamran A. Ahmed, Prakash Chinnaiyan, William J. Fulp, Steven Eschrich, Javier F. Torres-Roca, Jimmy J. Caudell

**Affiliations:** ^1^ Department of Radiation Oncology, H. Lee Moffitt Cancer Center and Research Institute, Tampa, FL 33612, USA; ^2^ Department of Biostatistics, H. Lee Moffitt Cancer Center and Research Institute, Tampa, FL 33612, USA; ^3^ Department of Bioinformatics, H. Lee Moffitt Cancer Center and Research Institute, Tampa, FL 33612, USA; ^4^ Department of Radiation Oncology, William Beaumont Hospital, Royal Oak, MI 48073, USA

**Keywords:** radiosensitivity, signature, glioblastoma

## Abstract

We have previously developed a multigene expression model of tumor radiosensitivity (RSI) with clinical validation in multiple cohorts and disease sites. We hypothesized RSI would identify glioblastoma patients who would respond to radiation and predict treatment outcomes. Clinical and array based gene expression (Affymetrix HT Human Genome U133 Array Plate Set) level 2 data was downloaded from the cancer genome atlas (TCGA). A total of 270 patients were identified for the analysis: 214 who underwent radiotherapy and temozolomide and 56 who did not undergo radiotherapy. Median follow-up for the entire cohort was 9.1 months (range: 0.04–92.2 months). Patients who did not receive radiotherapy were more likely to be older (*p* < 0.001) and of poorer performance status (*p* < 0.001). On multivariate analysis, RSI is an independent predictor of OS (HR = 1.64, 95% CI 1.08–2.5; *p* = 0.02). Furthermore, on subset analysis, radiosensitive patients had significantly improved OS in the patients with high MGMT expression (unmethylated MGMT), 1 year OS 84.1% vs. 53.7% (*p* = 0.005). This observation held on MVA (HR = 1.94, 95% CI 1.19–3.31; *p* = 0.008), suggesting that RT has a larger therapeutic impact in these patients. In conclusion, RSI predicts for OS in glioblastoma. These data further confirm the value of RSI as a disease-site independent biomarker.

## INTRODUCTION

Glioblastoma is the most common central nervous system malignancy in adults. Despite advances in surgical resection, chemotherapy, and radiation over the past several decades, glioblastoma continues to have a median survival of 14–15 months [1]. Current standard of care for patients with glioblastoma is a maximal safe resection followed by radiotherapy with concurrent and adjuvant temozolomide [1]. There are several biomarkers to help predict prognosis in patients following surgical resection. O(6)-Methylguanine-DNA methyltransferase (MGMT) gene silencing has been found to be prognostic and predictive for outcomes following surgery and temozolomide [2]. In addition, patients with mutations in the active site of isocitrate dehydrogenase 1 (IDH1) have improved outcomes over those with wild-type IDH1 [3]. As we move towards an era of personalized medicine, there is not yet a tool to help with the selection of patients most likely to benefit from radiotherapy and assist in dose selection in the management of glioblastoma.

There are known differences in radiosensitivity between different tumor types and between different patients with the same tumor type. We have previously developed a radiosensitivity index (RSI) modeled as a function of gene expression, tissue of origin, ras and p53 status correlated to the surviving fraction of cells at 2 Gy (SF2) in a panel of 48 human cancer cell lines [4]. This model predicts a radiosensitivity index (RSI) that is directly proportional to tumor radioresistance (RSI, high index = radioresistance). Prior work has shown RSI to be disease-site independent and predictive for clinical outcome in RT-treated patients in a number of different primary cancers including esophageal, rectal, head and neck, breast, prostate, pancreas, metastatic colon cancer, and non-small cell lung cancer (NSCLC) [5–11]. Herein, we hypothesized RSI would identify those patients with glioblastoma who would be more likely to respond to radiation treatment and therefore predict outcomes following standard adjuvant treatment.

## RESULTS

### Patient characteristics

Median follow-up for the entire cohort was 9.1 months (range: 0.04–92.2 months); 11.4 months in the radiotherapy and temozolomide group and 1.7 months in the no radiotherapy group. Median follow-up for living patients was 8.7 months (range: 2.9–92.2 months). Clinical characteristics for the entire cohort are presented in Table 1. Patients who did not receive radiotherapy were more likely to be older (*p* < 0.001) and of poorer performance status (*p* < 0.001). No differences were noted in extent of resection (*p* = 0.54) and MGMT expression (*p* = 0.45) between patients that received temozolomide with or without radiation (Table 1).

**Table 1:** Patient characteristics

	Total, *n* (%)	No RT, *n* (%)	RT + TMZ, *n* (%)	*p* value
**Age**				
**<50**	61 (22.6)	3 (5.4)	58 (27.1)	<0.001
**≥50**	209 (77.4)	53 (94.6)	156 (72.9)	
**Gender**				
**Male**	169 (62.6)	31 (55.4)	138 (64.5)	0.218
**Female**	101 (37.4)	25 (44.6)	76 (35.5)	
**Race**				
**White/Other**	251 (93.7)	52 (92.9)	199 (93.0)	>0.99
**Black**	15 (5.6)	3 (5.4)	12 (5.6)	
**Unknown**	4 (1.5)	1 (1.8)	3 (1.4)	
**Type of Surgery**				
**Tumor Resection**	228 (84.4)	49 (87.5)	179 (83.6)	0.54
**<Tumor Resection**	42 (15.6)	7 (12.5)	35 (16.4)	
**ECOG**				
**01**	158 (58.5)	7 (12.5)	151 (70.6)	<0.001
**24**	64 (23.7)	21 (37.5)	43 (20.1)	
**Unknown**	48 (17.8)	28 (50.0)	20 (9.3)	
**MGMT Expression**				
**Low**	135 (50.0)	31 (55.4)	104 (48.6)	0.453
**High**	135 (50.0)	25 (44.6)	110 (51.4)	
**RSI**				
**RS**	67 (24.8)	19 (33.9)	48 (22.4)	0.08
**RR**	203 (75.2)	37 (66.1)	164 (77.6)	

RT = Radiotherapy

TMZ = Temozolomide

ECOG = Eastern Cooperative Oncology Group Performance Status

MGMT = O(6)-Methylguanine-DNA methyltransferase

RSI = Radiosensitivity index

RS = Radiosensitive

RR = Radioresistant

Patients who received radiation and temozolomide were categorized as radiosensitive (*n* = 48; 22%) and radioresistant (*n* = 166; 78%). Patients categorized as radiosensitive were more likely to have high MGMT expression (*p* = 0.02). Otherwise, no other significant differences were seen between groups (Table 2).

**Table 2:** Characteristics of radiosensitive and radioresistant patients

	Radiosensitive, *n* (%)	Radioresistant, *n* (%)	*p* value
**Age**			
**<50**	16 (33.3)	42 (25.3)	0.27
**≥50**	32 (66.7)	124 (74.7)	
**Gender**			
**Male**	30 (62.5)	108 (65.1)	0.74
**Female**	18 (37.5)	58 (34.9)	
**Race**			
**White/Other**	45 (95.7)	154 (93.9)	>0.99
**Black**	2 (4.3)	10 (6.1)	
**Type of Surgery**			
**Tumor Resection**	42 (87.5)	137 (76.5)	0.51
**<Tumor Resection**	6 (12.5)	29 (17.5)	
**ECOG**			
**01**	37 (77.1)	114 (68.7)	0.13
**24**	5 (11.6)	38 (22.9)	
**Unknown**	6 (12.5)	14 (8.4)	
**MGMT Expression**			
**Low**	16 (33.3)	88 (53.0)	0.02
**High**	32 (66.7)	78 (47.0)	

ECOG = Eastern Cooperative Oncology Group Performance Status

MGMT = O(6)-Methylguanine-DNA methyltransferase

### RSI and survival outcomes

Univariate analysis of overall survival (OS) is displayed in Table 3. As expected, factors found to be associated with improved OS on univariate analysis in the radiotherapy and temozolomide group were age (*p* = 0.004), MGMT expression (*p* = 0.002), and Eastern Cooperative Oncology Group (ECOG) performance status (*p* = 0.01). RSI did not predict OS in univariate analysis (HR = 1.34, *p* = 0.14, RS vs. RR 1 yr OS 87% vs. 64.4%, *p* = 0.14). However as noted above, RSI-radiosensitive patients were more likely to have high expression of MGMT (*p* = 0.02), a poor prognostic biomarker. When accounting for known prognostic factors on multivariate analysis (MVA), radioresistance was an independent factor predicting for a decline in OS (HR = 1.64, 95% CI 1.08–2.5; *p* = 0.02) in patients treated with radiotherapy and temozolomide (Table 4). Other factors found to be significant on MVA included age (*p* = 0.03), MGMT expression (*p* = 0.0002), and ECOG performance status (*p* = 0.01). As expected, no differences in OS were seen between radiosensitive and radioresistant patients who did not receive radiotherapy (*p* = 0.682).

**Table 3:** Univariate analysis of overall survival in patients treated with RT and temozolomide

Variable	Hazard Ratio (95% CI)	*p*-value
**RSI RR vs. RS**	1.34 (0.91,1.98)	0.14
**MGMT Expression High vs. Low**	1.7 (1.23,2.35)	0.002
**Age 50+ vs. 50**	1.76 (1.2,2.59)	0.004
**ECOG 24 vs. 01**	1.73 (1.14,2.64)	0.01
**ECOG Unknown vs. 01**	1.23 (0.73,2.07)	0.43
**Gender Male vs. Female**	1.07 (0.76,1.51)	0.71
**Race Black vs. Asian/White**	1.01 (0.45,2.29)	0.98
**Race Unknown vs. Asian White**	1.53 (0.48,4.82)	0.47

CI = Confidence Interval

RT = Radiotherapy

RSI = Radiosensitivity index

RS = Radiosensitive

RR = Radioresistant

MGMT = O(6)-Methylguanine-DNA methyltransferase

ECOG = Eastern Cooperative Oncology Group Performance Status

**Table 4:** Multivariate analysis of overall survival in patients treated with RT and temozolomide

Variable	Hazard Ratio (95% CI)	*p*-value
**RSI: RR vs. RS**	1.64 (1.08,2.5)	0.02
**MGMT Expression: High vs. Low**	1.99 (1.39,2.9)	0.0002
**Age: 50+ vs. 50**	1.54 (1,2.31)	0.03
**ECOG: 24 vs. 01**	1.74 (1.12,2.69)	0.01
**ECOG: Unknown vs. 01**	1.05 (0.6,1.81)	0.87

CI = Confidence Interval

RSI = Radiosensitivity Index

RS = Radiosensitive

RR = Radioresistant

MGMT = O(6)-Methylguanine-DNA methyltransferase

ECOG = Eastern Cooperative Oncology Group Performance Status

**Figure 1:**
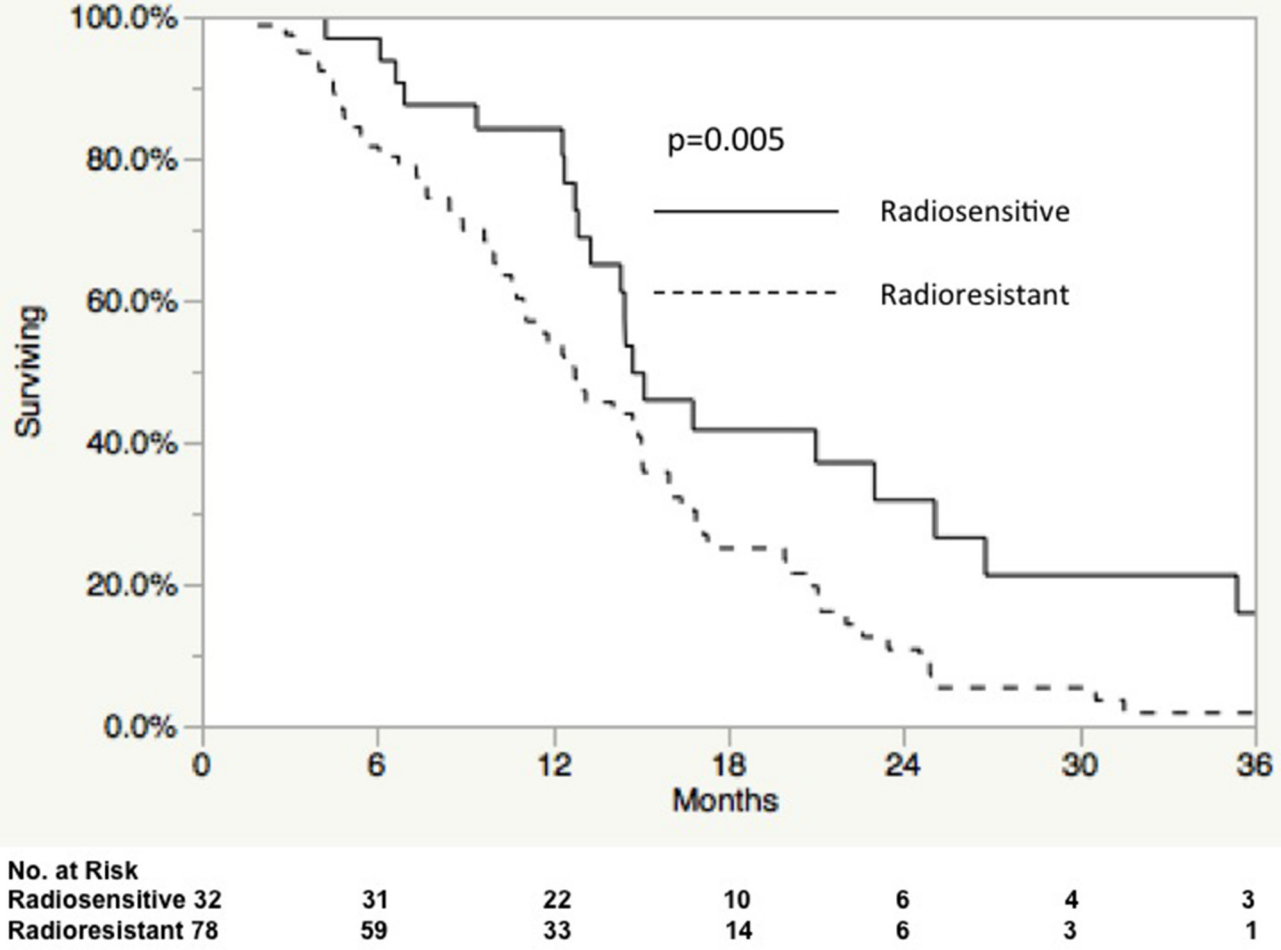
Overall survival in radioresistant and radiosensitive patients with high MGMT expression treated with radiation and temozolomide

The observation that increased radiosensitivity was more prevalent in patients with a poorer prognosis at baseline (MGMT-high expression) led us to hypothesize that a subset analysis could identify sub-populations where RSI had a larger predictive power. On stratification by MGMT expression, radiosensitive patients had significantly improved OS in the high MGMT group, 1 year OS 84.1% vs. 53.7% (*p* = 0.005; Figure 1). When restricting the analysis to patients with high MGMT expression, RSI was the strongest predictor of OS on MVA (HR = 1.94, 95% CI 1.19–3.31; *p* = 0.008), Table 5.

**Table 5:** Multivariate analysis of overall survival in MGMT high patients treated with RT and temozolomide

Variable	Hazard Ratio (95% CI)	*p*-value
**RSI: RR vs. RS**	1.94 (1.19,3.31)	0.008
**Age: 50+ vs. 50**	1.34 (0.8, 2.38)	0.29
**ECOG: 24 vs. 01**	1.41 (0.71,2.55)	0.31
**ECOG: Unknown vs. 01**	0.74 (0.4,1.36)	0.35

CI = Confidence Interval

RSI = Radiosensitivity Index

RS = Radiosensitive

RR = Radioresistant

## DISCUSSION

The development of biomarker based models to guide treatment algorithms is a central goal of personalized medicine. RSI is distinct from other biomarkers since it was developed to be radiation specific and disease site independent. In doing so, the RSI algorithm has been validated in multiple malignancies [5–11]. In these cohorts, we found the RSI algorithm correlated to clinical endpoints in patients treated with RT. Likewise, since RSI is related to SF2, an experimental measure of cellular radiosensitivity, we found the RSI algorithm did not predict outcomes in patients treated without RT [5, 6]. Here, we test RSI in a cohort of glioblastoma patients and find it to be an independent predictor of OS in glioblastoma patients treated with RT and temozolomide. On subset analysis, we found RSI had a larger predictive power in patients with high MGMT expression, suggesting RT may result in a larger therapeutic benefit in this sub-population.

Glioblastoma management remains a difficult treatment paradigm for practitioners with significant clinical nihilism. Results from large randomized trials attempting to improve outcomes via intensification of temozolomide [12] or the addition of bevacizumab [13, 14] were unable to show increased OS. In addition, studies have not shown improvement in OS with uniform radiation dose intensification [15]. Thus, recent studies have assessed whether a de-escalation of treatment may be adequate in some patient populations. In a group of patients’ age 70 or older with glioblastoma, Keime-Guibert et al. showed a modest absolute OS benefit of 12.2 weeks with the addition of radiotherapy to supportive care [16]. Wick et al. showed that in patients age 65 years or older with anaplastic astrocytoma or glioblastoma, temozolomide alone was non-inferior to radiotherapy alone; however, in MGMT unmethylated patients, radiotherapy was superior to temozolomide [17]. In our current study, we find the prognostic value of the RSI index to be greatest in patients with high MGMT expression. Since the benefit of RT over temozolomide is higher than in patients with low MGMT expression, we hypothesize the RSI index, which is an RT specific biomarker, may be particularly useful in providing prognostic information and potentially predictive information for dose escalation in this population. A recent SEER analysis of patients treated between 1973 and 2006 revealed a decline in the utilization of radiotherapy in the management of glioblastoma [18]. Thus, the appropriate identification of patients who will achieve maximal benefit from radiation is essential.

Several institutions have tested hypofractionation schedules for the adjuvant treatment of glioblastoma [19–25]. Terasaki et al. reported phase I results of 26 patients treated with 45 Gy in 15 fractions with concurrent and adjuvant temozolomide [25]. With a median follow-up of 20 months, the median OS was 15.6 months with acceptable toxicity. In addition, the University of Colorado performed a phase I hypofractionated trial to 60 Gy in postoperative cavities measuring ≤6 cm. Patients were treated with escalating doses of 3 Gy per fraction (3 patients) to 6 Gy per fraction (6 patients) with concurrent and adjuvant temozolomide. Median survival was 16.2 months with one patient treated in 4 Gy fractions experiencing vision loss in the left eye. Trials have also assessed dose escalation in glioblastoma [15, 26]. Chan et al. assessed dose escalation in 34 post-operative glioblastoma patients to 90 Gy with a 2 year OS of 12.9% [26].

The interest in hypofractionation and treatment de-intensification indicates the need for better tools to select those patients most likely or least likely to benefit from radiation. Although additional validation would be required, RSI could prove critical in identifying ideal sub-populations for dose optimization either via de-intensification or hypofractionation.

It is now believed there are three unique subclasses with prognostic implications in malignant glioma [27]. The three tumor subclasses termed proneural, proliferative, and mesenchymal were identified based on the dominant features of their respective gene signatures and are believed to recapitulate stages in the differentiation of neural stem cells, lending them biologic relevance. In the future, these subtypes may require different treatment algorithms both with adjuvant radiation and chemotherapy management. Next steps in our analysis would be to assess whether RSI can help predict treatment outcomes in these subtypes as well as its correlation with other proposed gene signatures in glioblastoma [28] as we move closer to an era when treatment decisions will be patient specific.

Although these initial findings are quite interesting and hypothesis generating, they are not without limitations. First, this is a retrospective analysis and there might be biases that may be impacting our results. Many clinical details are not available from the TCGA database and thus we are unable to assess all prognostic indicators in glioblastoma with RSI. Furthermore, we have presented validation in only one cohort and additional analysis in other datasets would be desirable. However, RSI is a fairly mature signature that has been tested in multiple disease sites and independent cohorts. In addition, the RSI algorithm and cutpoints were pre-defined, thus limiting error related to multiple testing. Although additional validation is necessary, in the present analysis we demonstrate RSI predicts for OS on MVA in glioblastoma patients treated with RT and temozolomide. This further confirms the value of RSI as a disease site-independent clinical biomarker.

## MATERIALS AND METHODS

### TCGA data

Clinical and array based gene expression (Affymetrix HT Human Genome U133 Array Plate Set) level 2 data was downloaded from the cancer genome atlas (TCGA). Protocols have previously been described [29]. Data from 574 glioblastoma samples is available in the TCGA database. Patient data was included for analysis if gene expression array data was available with a sample that included ≥50% tumor. Patients who underwent treatment with radiotherapy and concurrent temozolomide or no radiotherapy were included. Additional exclusion criteria were neoadjuvant treatment, radiotherapy initiated ≥6 months after pathological diagnosis, or missing follow-up data. Therefore, 270 patients remained for analysis: 214 who underwent radiotherapy and temozolomide and 56 who did not undergo radiotherapy. Initial pathologic diagnosis of patients included in the study was between 1993 and 2011 with 93% of patients treated in 2002 or afterwards. In the 214 patients that received radiation and temozolomide, median radiation dose was 60 Gy (range: 12.6 −97 Gy). Five patients were treated with radiation doses below 45 Gy with the majority of patients (*n* = 159; 74%) treated with 60 Gy. Four patients in the no radiotherapy group received temozolomide.

### Radiosensitivity index

Probesets utilized for each gene were the same as in prior studies [4–6, 30]. RSI was determined using the previously published rank-based linear regression algorithm:

RSI = −.0098009 * AR + 0.0128283 * cJun+ 0.0254552 * STAT1 − 0.0017589 * PKC − 0.0038171 * RelA + 0.1070213 * cABL − 0.0002509 * SUMO1 − 0.0092431 * PAK2 − 0.0204469 * HDAC − 0.0441683 * IRF1.

As previously done, the 25th percentile for RSI was pre-defined as the cut-point to dichotomize patients into radiosensitive and radioresistant groups [5, 6]. The 25th percentile RSI value for dichotomization was 0.54 (range 0.14–0.96).

### O(6)-Methylguanine-DNA methyltransferase (MGMT)

We utilized Probe 204879_at, which maps to the MGMT gene for expression analysis. Others have previously shown that MGMT mRNA expression is correlated with MGMT promoter methylation [31]. In the minority of patients where discordant findings were seen, MGMT mRNA expression was a better predictor of outcome compared to MGMT promoter methylation [31]. Thus, in this analysis the median MGMT expression value was used to dichotomize patients into low and high expression groups. Median MGMT expression level was 5.44 (range 3.59–7.99).

### Statistical analyses

OS was defined as time from pathological diagnosis, last follow-up, or death. Fisher's exact test with Monte Carlo simulation was used to study association between radiosensitive and radioresistant variables and other potential prognostic variables. Kaplan-Meier survival curves for OS were fit for the radiosensitive and radioresistant groups and difference tested via log-rank. Multivariate Cox proportional hazard models were used to select potential predictors for OS. All analyses were done in SPSS (version 19), tests were two sided, and had a significance level of 0.05.
